# Supplementary material for: On the predictability of infectious disease outbreaks
Source: Nat Commun. 2019 Feb 22;10:898. doi: 10.1038/s41467-019-08616-0 (PMC6385200; doi:10.1038/s41467-019-08616-0)
Supplement: Supplementary file 1 — Supplementary Information [file 41467_2019_8616_MOESM1_ESM.pdf]

Supplementary Information for “On the predictability of infectious disease outbreaks”

Samuel V. Scarpino[1,2,3,4,5,6†,*] **and Giovanni Petri**[6,7,†,**]

[1]Network Science Institute, Northeastern University, Boston, MA, 02115, USA
[2]Marine & Environmental Sciences, Northeastern University, Boston, MA, 02115, USA
[3]Physics, Northeastern University, Boston, MA, 02115, USA
[4]Health Sciences, Northeastern University, Boston, MA, 02115, USA
[5]Dharma Platform, Washington, DC, 20005, USA
[6]ISI Foundation, 10126 Turin, Italy
[7]ISI Global Science Foundation, New York, NY 10018, USA
[†]Both authors contributed equally to this work.
[*]s.scarpino@northeastern.edu
[**]giovanni.petri@isi.it

Additional data and code are available at: **`https://github.com/Emergent-Epidemics/infectious_disease_predictability`**

Supplementary Methods

Permutation Entropy: Here, we make use of *permutation entropy* as a model-independent measure of the growth in complexity and unpredictability of infectious disease time series. Given a time series $\{x_t\}_{t=1,\dots,N}$ indexed by positive integers, an embedding dimension d and a temporal delay τ , one can consider the set of all sequences of values s of the type $s = \{x_t, x_{t+\tau}, \dots, x_{t+(d-1)\tau}\}$. Note that successive values $x_{t+i\tau}, x_{t+(i+1)\tau}$ for generic i can be in an arbitrary relative order. To each s, one can associates the permutation π of order d that makes s totally ordered, that is $\tilde{d} = \pi(d) = \{x_{t_i}, \dots, x_{t_N}\}$ such that $x_{t_i} < x_{t_j} \forall t_i < t_j$. In this way, via π we associate a rank-order quantity that is independent of the actual values the timeseries takes and we can associate a probability p_π to each permutation by simply counting how many times if appears in the data as compared to the total number of sequences appearing. The permutation entropy of time series $\{x_t\}$ is then given by the Shannon entropy on the permutation orders, that is $H^p_{d,\tau}(\{x_t\}) = -\sum_\pi p_\pi \log p_\pi$. In Figure **??** we provide an explicit example of the computation of the permutation entropy. In order to compare across

different dimensions d, we normalize the entropies by the (log)number of observed symbols. We find that diseases cluster based on the best-fit dimension, d, see Figure 2, and that the disease specific slopes for a random effects model of (log)entropy and (log)timeseries can be predicted based on the embedding dimension 3.

Weighted Permutation Entropy: In the manuscript, we show results obtained by fixing $\tau = 1$ to aid the intuition of the reader and select the most conservative (smallest) value of $H^p(\{x_t\}) = \min_d H^p_{d,\tau=1}(\{x_t\})$ by swiping over a wide range of possible d values. However, the qualitative results do not change even when we allow for a full swipe on (d,τ) pairs and setting $H^p(\{x_t\}) = \min_{d,\tau} H^p_{d,\tau}(\{x_t\})$, see Figure 4. In addition, we also confirmed that similar results were obtained by using the weighted permutation entropy, as presented in [1,2] and implemented in the R package statcomp v. 0.0.1.1000[3], see Figure 5. Although, it's worth pointing out that weighted permutation entropy is attempting to normalize away exactly the kind of structure infectious disease modellers aim to predict.

Markov chain simulations: In order to assess the amount of non-random structure in the real outbreak time series, we build synthetic symbolic time series by simulating Markov chains over the symbol distributions obtained from the empirical time series. For each real time series $\{x_t\}_i$, we extract the set of permutation symbols $\{\pi\}$ as in the standard calculation for permutation entropy. We utilize $\tau = 1$ and the embedding dimension d_i previously selected during the permutation entropy computation as described in Brandmeier (2015)[4]. For a time series with embedding dimension d, there are a maximum number of $d!$ states, corresponding to the possible permutations of length d. Using the permutations as states, we then count the number of transitions n_{ij} in the real time series between each pair of symbols (i,j) and use it to build a Markov chain with transition probabilities between states given by $p_{ij} = \frac{n_{ij}}{\sum_j n_{ij}}$. In order to obtain a synthetic symbolic series, we repeatedly start from a randomly selected node and use the Markov Chain described above to produce symbolic series with the same number of symbols as the corresponding real time series. For each iteration, we calculate the associated symbolic entropy. In Figure 6 we compare the synthetic entropies versus the

permutation entropy of the original time series and show that the former are systematically higher than the real ones, implying that there is additional structure in the outbreak time series that is not captured simply by the probabilistic transition structure.

1 Supplementary Figures

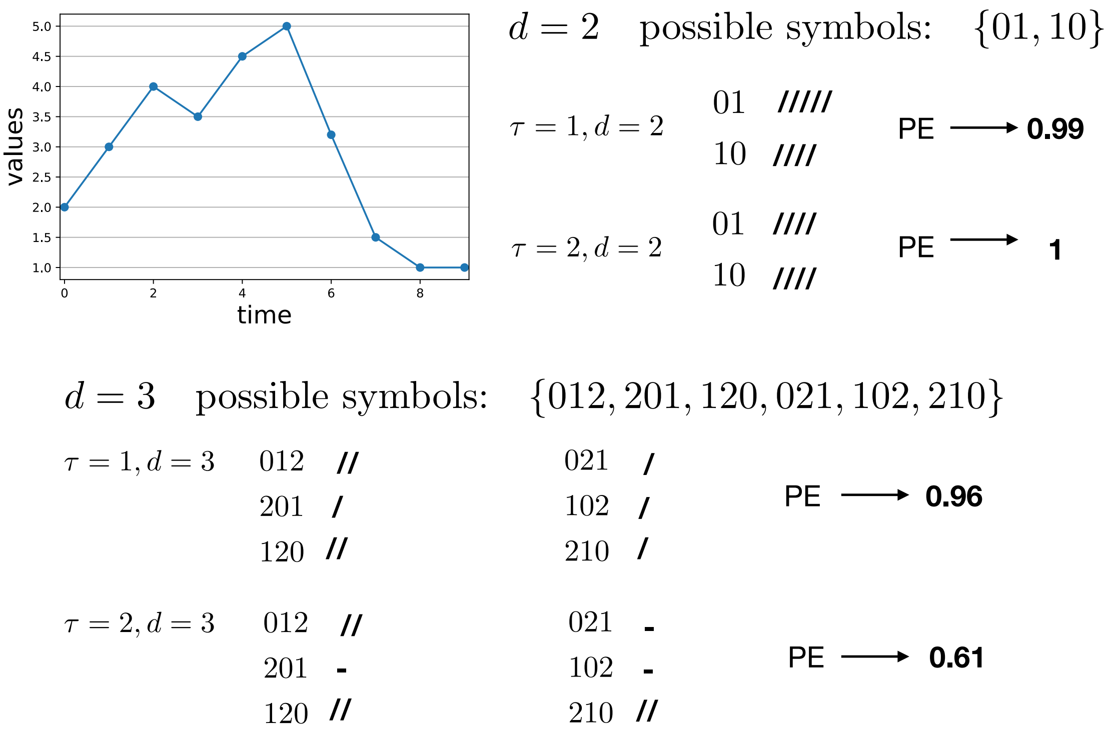

Supplementary Figure 1. Computation of permutation entropy. We show how the computation is performed for a toy signal for two different embedding dimensions d and delays τ. The dashed by each possible symbol (permutation ordering) correspond to the how many time that symbols is observed in the time series.

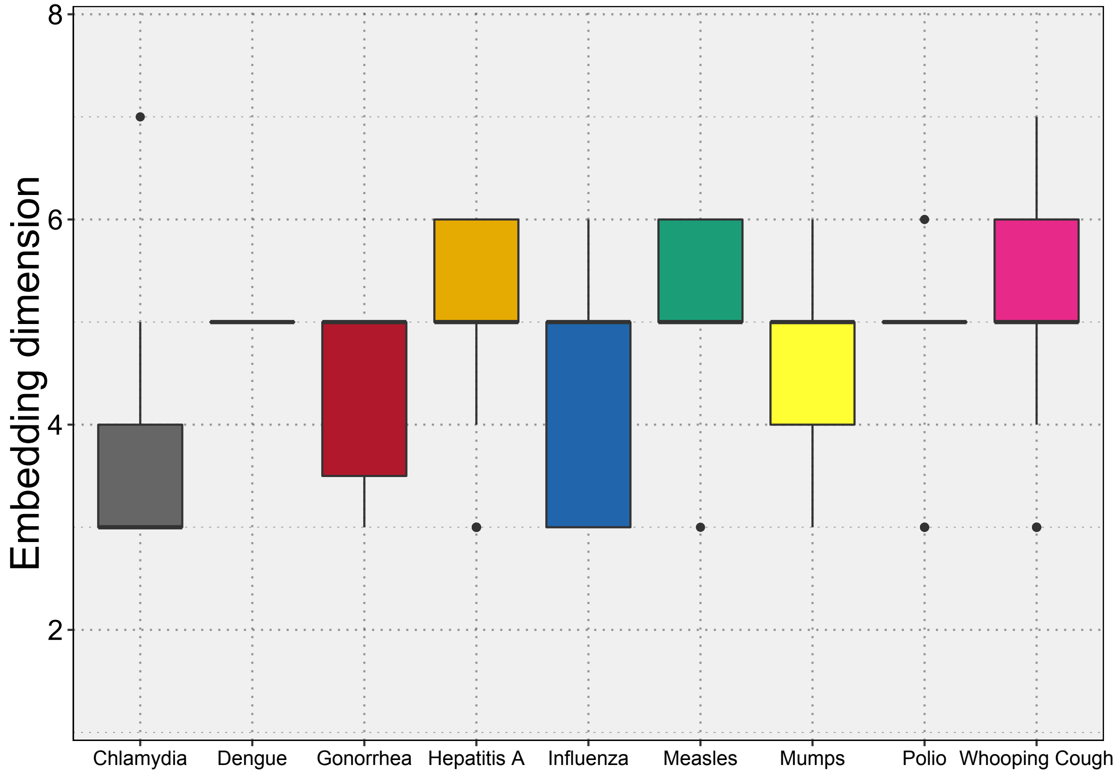

Supplementary Figure 2. Embedding dimensions. We show the distributions of embedding dimensions d by disease. Embedding dimensions were obtained for each disease independently by minimizing the permutation entropy over a wide range of potential dimensions ($d \in [1,20]$). Making this conservative choice on d allow us to interpret this length of the fundamental symbols used in computing permutation entropy as the natural temporal scale for the predictability of the corresponding timeseries. Notably, all diseases display narrow distributions and peaks between 3 and 6 weeks, with STDs, influenza and mumps being characterized by the shortest entropy horizons. Boxes represent 25th to 75th percentiles of the distributions, whiskers 5th to 95th percentiles

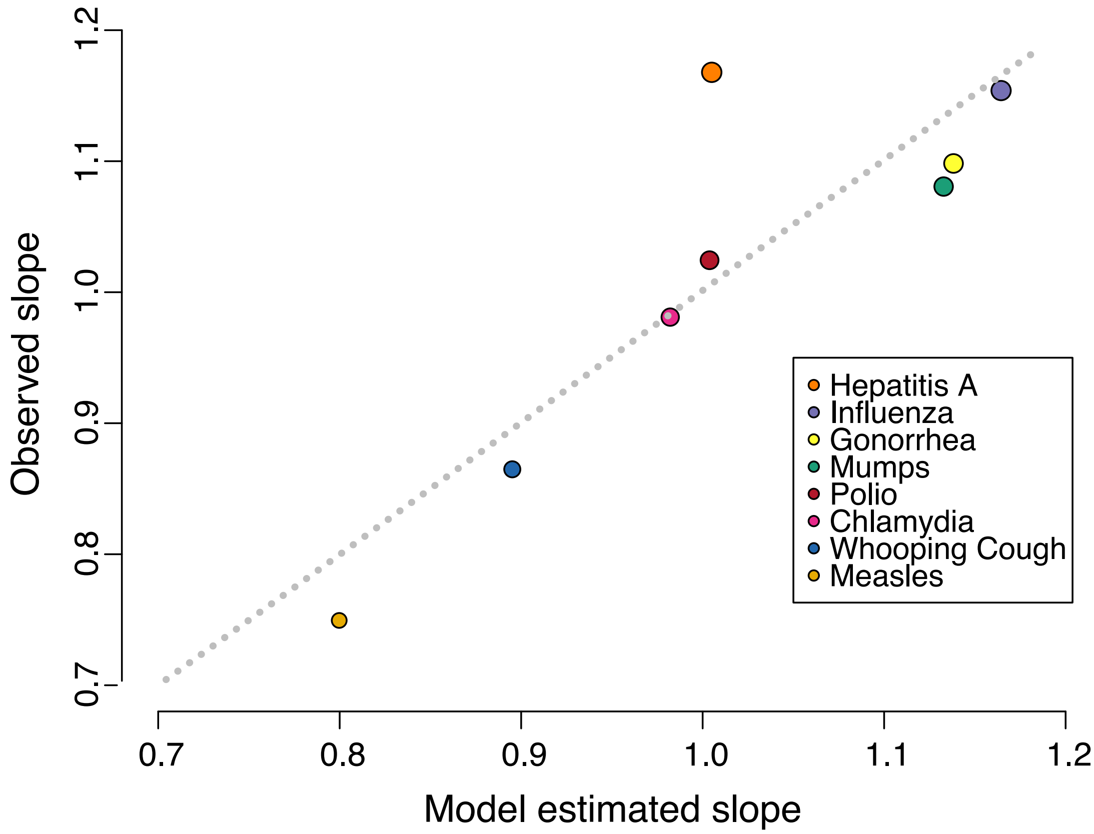

Supplementary Figure 3. Slope of H^p growth. In the main text we show that a mixed effect model yields a linear relationship between the (log)entropy and (log)timeseries length, where disease has a random effect on the slope. We find that the disease specific slopes, i.e. the fixed effect slope plus the average random effect for each disease, can be predicted using only the embedding dimension, giving additional support to d as a fundamental dynamical feature of the underlying spreading process.

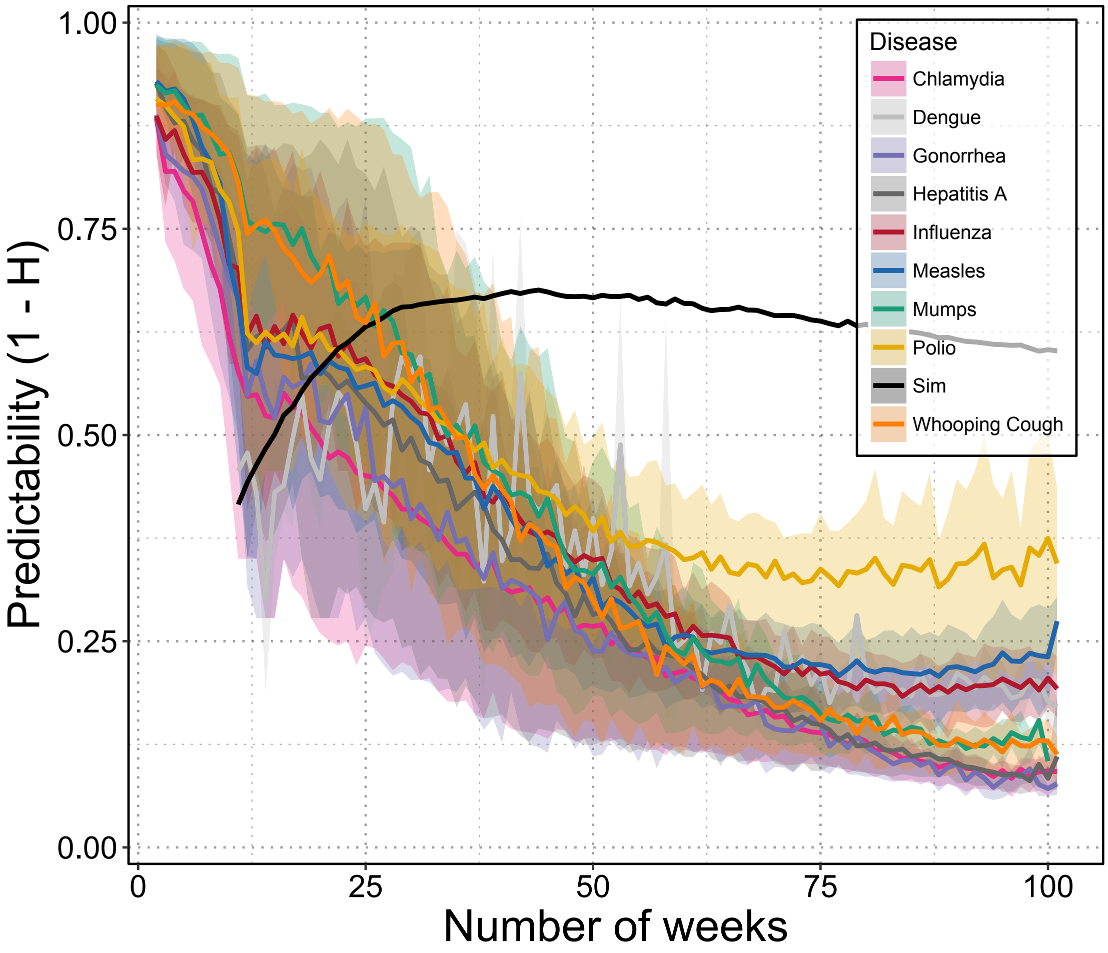

Supplementary Figure 4. Single outbreaks are often predictable based on sweeping across a range for both the dimension d and the time-delay τ. The average predictability $(1 - H^p)$ for weekly, state-level data from nine diseases is plotted as a function of time series length in weeks. We optimized, i.e. find the minimum PE, sweep across range of dimensions $d = 1 - 7$ weeks and time delays $\tau = 1 - 12$ weeks. Then, for each disease, we selected 1,000 random starting locations in each time series and calculated the weighted permutation entropy in rolling windows in lengths ranging from 2 to 104 weeks. The solid lines indicate the mean value and the shaded region marks the interquartile range across all states and starting locations in the time series. Although the slopes are different for each disease, in all cases, longer time series result in lower predictability. However, most diseases are predictable across single outbreaks and disease time series cluster together, i.e. there are disease-specific slopes on the relationship between predictability and time series length. To aid in interpretation, the black line plots the median permutation entropy across 20,000 stochastic simulations of a Susceptible Infectious Recovered (SIR) model, as described in the Supplement. This SIR model would be considered "predictable," thus values above the black-line might be thought of as in-the-range where model-based forecasts are expected to outperform forecasts based solely on statistical properties of the time series data.

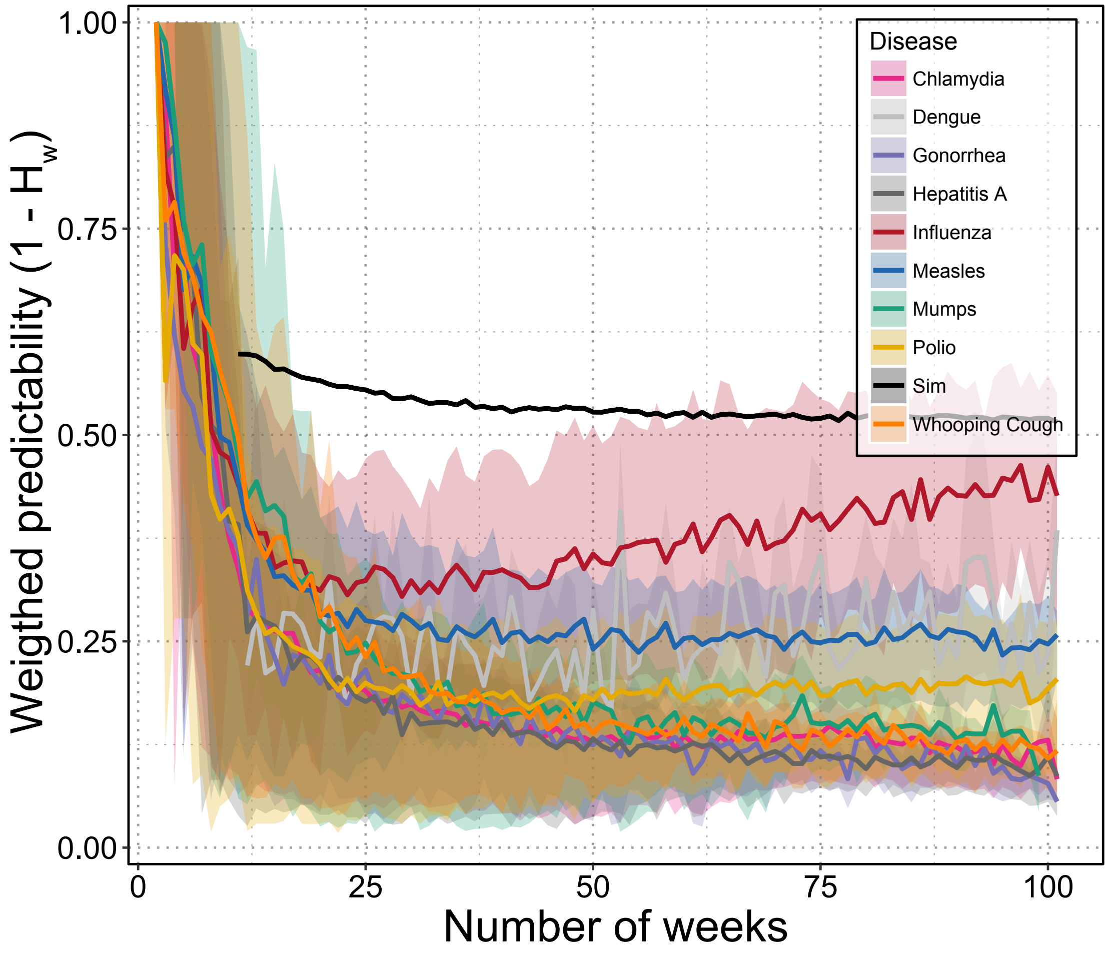

Supplementary Figure 5. Single outbreaks are often predictable based on weighted PE. The average weighted predictability $(1 - H_w^p)$ for weekly, state-level data from nine diseases is plotted as a function of time series length in weeks; where H_w^p is the weighted permutation entropy following[1,2]. For each disease, we selected 1,000 random starting locations in each time series and calculated the weighted permutation entropy in rolling windows in lengths ranging from 2 to 104 weeks. The solid lines indicate the mean value and the shaded region marks the interquartile range across all states and starting locations in the time series. Although the slopes are different for each disease, in all cases, longer time series result in lower predictability. However, most diseases are predictable across single outbreaks and disease time series cluster together, i.e. there are disease-specific slopes on the relationship between predictability and time series length. To aid in interpretation, the black line plots the median permutation entropy across 20,000 stochastic simulations of a Susceptible Infectious Recovered (SIR) model, as described in the Supplement. This SIR model would be considered "predictable," thus values above the black-line might be thought of as in-the-range where model-based forecasts are expected to outperform forecasts based solely on statistical properties of the time series data.

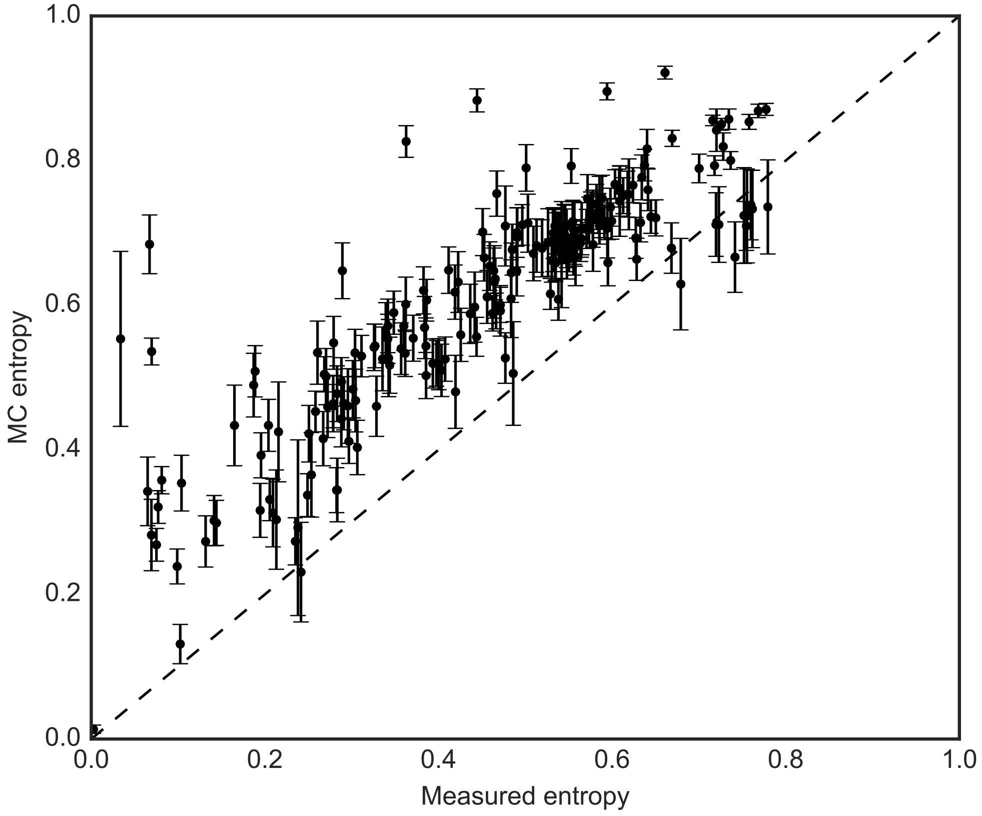

Supplementary Figure 6. ***d*-th order Markov Chain entropy.** We simulated Markov Chains on the symbol codebooks extracted for each timeseries and we show that for all timeseries the estimated H^p is higher or comparable to the one computed from data. For clarity, we show here only the results for a selection of timeseries (with length between 300-1000 weeks) but the results apply to all series. Error bars represent 95% confidence intervals.

2 Supplementary Tables

Disease	Mean R_0	Range	Citation(s)
chlamydia	0.99	(0.43 – 1.49)	[5,6,7,8]
gonorrhea	1.34	(0.82 – 2.0)	[6,9,10]
hepatitis A	2.45	(0.40 – 4.0)	[11,12,13]
influenza	1.47	(0.9 – 2.1)	[14,15]
measles	15.10	(4.7 – 31.0)	[16,17,18,19]
mumps	9.94	(3.0 – 31.5)	[20,21]
polio	5.36	(4.0 – 7.0)	[16,22,23]
whooping cough	14.75	(5 – 20)	[16,22,24,25,26]
Zika	2.7	(0.50 – 6.3)	[27]

Supplementary Table 1. Values of the basic reproductive ratio (R_0) for diseases included in this study were determined via literature review.

Supplementary References

1. Fadlallah, B., Chen, B., Keil, A. & Príncipe, J. Weighted-permutation entropy: A complexity measure for time series incorporating amplitude information. *Physical Review E* **87**, 022911 (2013).

2. Garland, J., James, R. & Bradley, E. Model-free quantification of time-series predictability. *Physical Review E* **90**, 052910 (2014).

3. Sippel, S., Lange, H. & Gans, F. *statcomp: Statistical Complexity and Information Measures for Time Series Analysis* (2016). URL `https://CRAN.R-project.org/package=statcomp`, r package version 0.0.1.1000.

4. Brandmaier, A. M. pdc: An R package for complexity-based clustering of time series. *Journal of Statistical Software* **67**, 1–23 (2015).

5. Potterat, J. J. *et al.* Chlamydia transmission: concurrency, reproduction number, and the epidemic trajectory. *American Journal of Epidemiology* **150**, 1331–1339 (1999).

6. Brunham, R. C., Nagelkerke, N. J., Plummer, F. A. & Moses, S. Estimating the basic reproductive rates of Neisseria gonorrhoeae and Chlamydia trachomatis: the implications of acquired immunity. *Sexually transmitted diseases* **21**, 353–356 (1994).

7. Althaus, C. L., Choisy, M. & Alizon, S. How sex acts scale with the number of sex partners: evidence from Chlamydia trachomatis data and implications for control. *PeerJ PrePrints* **3**, e1821 (2015).

8. Liu, F. *et al.* Assessment of transmission in trachoma programs over time suggests no short-term loss of immunity. *PLoS Negl Trop Dis* **7**, e2303 (2013).

9. McCluskey, C. C., Roth, E. & Van Den Driessche, P. Implication of Ariaal sexual mixing on gonorrhea. *American journal of human biology* **17**, 293–301 (2005).

10. Fingerhuth, S. M., Bonhoeffer, S., Low, N. & Althaus, C. L. Antibiotic-resistant Neisseria gonorrhoeae spread faster with more treatment, not more sexual partners. *PLoS Pathog* **12**, e1005611 (2016).

11. Regan, D. *et al.* Estimating the critical immunity threshold for preventing hepatitis A outbreaks in men who have sex with men. *Epidemiology and infection* **144**, 1528–1537 (2016).

12. Gay, N., Morgan-Capner, P., Wright, J., Farrington, C. & Miller, E. Age-specific antibody prevalence to hepatitis A in England: implications for disease control. *Epidemiology and infection* **113**, 113 (1994).

13. Van Effelterre, T. P., Zink, T. K., Hoet, B. J., Hausdorff, W. P. & Rosenthal, P. A mathematical model of hepatitis a transmission in the United States indicates value of universal childhood immunization. *Clinical infectious diseases* **43**, 158–164 (2006).

14. Pourbohloul, B. *et al.* Initial human transmission dynamics of the pandemic (H1N1) 2009 virus in North America. *Influenza and other respiratory viruses* **3**, 215–222 (2009).

15. Chowell, G., Miller, M. & Viboud, C. Seasonal influenza in the United States, France, and Australia: transmission and prospects for control. *Epidemiology and infection* **136**, 852–864 (2008).

16. Anderson, R. M., May, R. M. & Anderson, B. *Infectious diseases of humans: dynamics and control*, vol. 28 (Wiley Online Library, 1992).

17. Wichmann, O. *et al.* Large measles outbreak at a German public school, 2006. *The Pediatric infectious disease journal* **26**, 782–786 (2007).

18. van Boven, M. *et al.* Estimation of measles vaccine efficacy and critical vaccination coverage in a highly vaccinated population. *Journal of the Royal Society Interface* **7**, 1537–1544 (2010).

19. Grais, R. F. *et al.* Estimating transmission intensity for a measles epidemic in Niamey, Niger: lessons for intervention. *Transactions of the Royal Society of Tropical Medicine and Hygiene* **100**, 867–873 (2006).

20. Whitaker, H. & Farrington, C. Estimation of infectious disease parameters from serological survey data: the impact of regular epidemics. *Statistics in medicine* **23**, 2429–2443 (2004).

21. Kanaan, M. & Farrington, C. Matrix models for childhood infections: a Bayesian approach with applications to rubella and mumps. *Epidemiology and Infection* **133**, 1009–1021 (2005).

22. Plotkin, S. A., Orenstein, W. A. & Offit, P. A. *Vaccines (Sixth Edition)* (W.B. Saunders, 2013).

23. Tebbens, R. J. D. & Thompson, K. M. Modeling the potential role of inactivated poliovirus vaccine to manage the risks of oral poliovirus vaccine cessation. *Journal of Infectious Diseases* **210**, S485–S497 (2014).

24. Kretzschmar, M., Teunis, P. F. & Pebody, R. G. Incidence and reproduction numbers of pertussis: estimates from serological and social contact data in five European countries. *PLoS Med* **7**, e1000291 (2010).

25. Althouse, B. M. & Scarpino, S. V. Asymptomatic transmission and the resurgence of Bordetella pertussis. *BMC medicine* **13**, 146 (2015).

26. de Cellès, M. D., Magpantay, F. M., King, A. A. & Rohani, P. The pertussis enigma: reconciling epidemiology, immunology and evolution. In *Proc. R. Soc. B*, vol. 283, 20152309 (The Royal Society, 2016).

27. Gao, D. *et al.* Prevention and control of Zika as a mosquito-borne and sexually transmitted disease: a mathematical modeling analysis. *Scientific reports* **6** (2016).
