# Supplementary material for: On the predictability of infectious disease outbreaks
Source: Nat Commun. 2019 Feb 22;10:898. doi: 10.1038/s41467-019-08616-0 (PMC6385200; doi:10.1038/s41467-019-08616-0)
Supplement: Supplementary file 3 — Reporting Summary [file 41467_2019_8616_MOESM3_ESM.pdf]

nature research

Corresponding author(s): Samuel V. Scarpino

Last updated by author(s): Jan 3, 2019

Reporting Summary

Nature Research wishes to improve the reproducibility of the work that we publish. This form provides structure for consistency and transparency in reporting. For further information on Nature Research policies, see Authors & Referees and the Editorial Policy Checklist.

Statistics

For all statistical analyses, confirm that the following items are present in the figure legend, table legend, main text, or Methods section.

n/a	Confirmed	
☐	☒	The exact sample size (n) for each experimental group/condition, given as a discrete number and unit of measurement
☒	☐	A statement on whether measurements were taken from distinct samples or whether the same sample was measured repeatedly
☐	☒	The statistical test(s) used AND whether they are one- or two-sided<br>*Only common tests should be described solely by name; describe more complex techniques in the Methods section.*
☐	☒	A description of all covariates tested
☐	☒	A description of any assumptions or corrections, such as tests of normality and adjustment for multiple comparisons
☐	☒	A full description of the statistical parameters including central tendency (e.g. means) or other basic estimates (e.g. regression coefficient) AND variation (e.g. standard deviation) or associated estimates of uncertainty (e.g. confidence intervals)
☐	☒	For null hypothesis testing, the test statistic (e.g. F, t, r) with confidence intervals, effect sizes, degrees of freedom and P value noted<br>*Give P values as exact values whenever suitable.*
☒	☐	For Bayesian analysis, information on the choice of priors and Markov chain Monte Carlo settings
☒	☐	For hierarchical and complex designs, identification of the appropriate level for tests and full reporting of outcomes
☐	☒	Estimates of effect sizes (e.g. Cohen's d, Pearson's r), indicating how they were calculated

Our web collection on statistics for biologists contains articles on many of the points above.

Software and code

Policy information about availability of computer code

Data collection	All code and data associated with this study can be found here: https://github.com/Emergent-Epidemics/infectious_disease_predictability.
Data analysis	All code and data associated with this study can be found here: https://github.com/Emergent-Epidemics/infectious_disease_predictability.

For manuscripts utilizing custom algorithms or software that are central to the research but not yet described in published literature, software must be made available to editors/reviewers. We strongly encourage code deposition in a community repository (e.g. GitHub). See the Nature Research guidelines for submitting code & software for further information.

Data

Policy information about availability of data

All manuscripts must include a data availability statement. This statement should provide the following information, where applicable:

- Accession codes, unique identifiers, or web links for publicly available datasets
- A list of figures that have associated raw data
- A description of any restrictions on data availability

Empirical data for all diseases--aside from dengue--were obtained from the U.S.A. National Notifiable Diseases Surveillance System as digitized by Project Tycho. Dengue data were obtained from the Pandemic Prediction and Forecasting Science and Technology Interagency Working Group under the National Science and Technology Council. All code and data associated with this study can be found here: https://github.com/Emergent-Epidemics/infectious_disease_predictability.

Field-specific reporting

Please select the one below that is the best fit for your research. If you are not sure, read the appropriate sections before making your selection.

☐ Life sciences ☐ Behavioural & social sciences ☒ Ecological, evolutionary & environmental sciences

For a reference copy of the document with all sections, see nature.com/documents/nr-reporting-summary-flat.pdf

Ecological, evolutionary & environmental sciences study design

All studies must disclose on these points even when the disclosure is negative.

Study description	To investigate the question of outbreak prediction, we study the information theoretic limits to forecasting across a broad set of infectious diseases using permutation entropy as a model independent measure of predictability. Studying the predictability of a diverse collection of historical outbreaks--including, chlamydia, dengue, gonorrhea, hepatitis A, influenza, measles, mumps, polio, and whooping cough--we identify a fundamental entropy barrier for infectious disease time series forecasting. However, we find that for most diseases this barrier to prediction is often well beyond the time scale of single outbreaks, implying prediction is likely to succeed.
Research sample	All available state-level data from Project Tycho were collected. https://www.tycho.pitt.edu/
Sampling strategy	No sub-sampling was performed. We performed simulation studies to determine that time series were of sufficient length to estimate permutation entropies.
Data collection	Empirical data for all diseases--aside from dengue--were obtained from the U.S.A. National Notifiable Diseases Surveillance System as digitized by Project Tycho. Dengue data were obtained from the Pandemic Prediction and Forecasting Science and Technology Interagency Working Group under the National Science and Technology Council. All code and data associated with this study can be found here: https://github.com/Emergent-Epidemics/infectious_disease_predictability.
Timing and spatial scale	Start and stop dates for collection varied by disease based on their availability in Project Tycho. https://www.tycho.pitt.edu/
Data exclusions	No data were excluded from this study
Reproducibility	All code and data associated with this study can be found here: https://github.com/Emergent-Epidemics/infectious_disease_predictability.
Randomization	We controlled for state as a covariate. No other randomization was performed.
Blinding	No blinding was performed.

Did the study involve field work? ☐ Yes ☒ No

Reporting for specific materials, systems and methods

We require information from authors about some types of materials, experimental systems and methods used in many studies. Here, indicate whether each material, system or method listed is relevant to your study. If you are not sure if a list item applies to your research, read the appropriate section before selecting a response.

Materials & experimental systems

n/a	Involved in the study
☒	☐ Antibodies
☒	☐ Eukaryotic cell lines
☒	☐ Palaeontology
☒	☐ Animals and other organisms
☒	☐ Human research participants
☒	☐ Clinical data

Methods

n/a	Involved in the study
☒	☐ ChIP-seq
☒	☐ Flow cytometry
☒	☐ MRI-based neuroimaging
